# Necrosis of the Jaw from Decayed Tooth

**Published:** 1866-01

**Authors:** 


Necrosis of the Jaw from Decayed Tooth.—Man, 47. Here there is a fistulous opening about midway between the angle and the symphisis of the lower jaw. This man has had this disease for two or three years, and it came from a diseased tooth. Was an alveolar abscess in the first place; these abscesses are often productive of a great deal of trouble. I have seen them extending as far down as the clavicle, and cured promptly by the removal of the tooth. They are very often improperly treated; caustics are applied and stimulating applications, but they are useless so long as the cause of the malady remains. This presents no malignant character. On introducing the probe we touch the alveolar process.

These abscesses often follow disease of the teeth—the tooth becomes a foreign body and keeps up the irritation. The pus often makes its way into the mouth and no evil results; at other times it opens through the face and leaves a fistulous opening. It seems strange that a dead tooth should produce such an amount of injury, but as the tooth is dead it acts as a foreign substance does in any other part of the body. We will extract the tooth, which is so much decayed as to render extraction difficult; we will also remove all portions of dead bone. There is a carious surface left, but we will inject with acid. muriat. gtt x. aquæ f.ʒj. to dissolve the earthy matter. The last two molars are diseased, and I shall order their removal.—*Med. and Sur. Reporter.*

